# Review of " *Parasites of medical and veterinary importance *" by Dietmar Steverding

**DOI:** 10.1186/1756-3305-4-170

**Published:** 2011-09-09

**Authors:** Geoff Hide

**Affiliations:** 1 Centre for Parasitology and Disease, School of Environment and Life Sciences, University of Salford, Salford, M5 4WT, UK

## Review

In the preface, this book is billed as a reference book of parasites of medical and veterinary importance. It also states that it lists over 600 parasites species providing an identical diet of information on each species in tabular form with a relatively small amount of free text introducing the tables. First impressions suggest it to be a very good reference book containing a comprehensive set of, easily accessed, information on a broad range of parasites. To get straight to the point, having read the book, the first impressions are accurate.

The book starts with a brief introduction - free text - to the broad groups of parasites, impact on humans and sources of nomenclature. By page 5 the book is straight onto the parasite groups where it is divided into three main sections, "Protozoa", "Metazoa" and "Micropredators as vectors for pathogens" followed by a series of indexes and annexes. Each section has a short introduction and then further broken down. For example, the protozoa are divided into 5 sections: Sarcomastigophora, Apicomplexa, Ciliophora, Microspora and "protozoa of uncertain classification". Purists of taxonomy might quibble with the groupings used but all section names will be familiar to the majority of users of this book. It is these sections that form the backbone of the book.

Each section comprises tables that describe a group of related parasites. For example, Table 4 includes the important genera *Trypanosoma *and *Leishmania *in the Kinetoplastida but also less familiar ones (e.g. *Cryptobia*). Similarly, in the metazoan section, Table 13 lists important species of Digenea such as *Schistosoma *and *Fasciola *but also less familiar parasites such as *Parastrigea*. Detailed inspection of the tables reveals two major challenges that the author must have endured. Which species to include? Which species are currently recognised as such in the ever changing world of taxonomy? The key parasites that have human and animal health importance are all there (as far as I can tell) and for that reason alone the book is highly valuable. The challenge, of course, is at the interface of what is and what is not important. There will be some readers whose "pet" parasite is missing. For example, I spotted two missing entries that might or might not be considered important: *Trypanosoma godfreyi *- a putative causative agent of trypanosomiasis in pigs - and *Plagiorchis spp. - *a genus of trematodes that infects a wide range of animal species (and occasionally humans). Should these and other such parasites be included? Are they of sufficient medical or veterinary importance? Do we know enough about their pathogenicity to decide on their importance? Similarly, a recent explosion in newly described or renamed species of *Cryptosporidium *- some of which are universally recognised and others not - must have created challenges as to what to include and perhaps even what to call them. I'm sure that Dr Steverding must have lost many hours of sleep over these issues! He does, however, guide us through this minefield with list of synonyms where they are reasonably established. Although I have raised the issue of inclusion, the choice of parasites in this book will not create great waves of discontent in the scientific community. The book is a monumental exercise in collating a detailed set of information on an astonishing range of parasites.

So what of the detail provided? Each table has 7 columns: "Species", "Size", "Host;Habitat", "Disease", "Symptoms", "Transmission" and "Distribution". Again the choice of what to and what not to include must have resulted in a further headache for the author! For my part the headings cover the key information and in sufficient depth.

What of the quality of the information in the book? I cannot judge this across the entire book but I sampled sections I knew about and some I knew less well and had to research. In the former category I found myself initially to be overly critical - as I had not clarified my expectations of the purpose of the book. I settled on the view that the book provided a good (and equivalent) level of detail on all species and that what was covered in the main sections was accurate and of good quality.

So what of the final provision of indexing and annexing? There are two indexes - subject and species - which I used extensively. An important bonus is the useful list of parasites found in different host species (from humans through cattle and fish to honey bees). The parasites included are the same as those included in the main body of the book.

Is this book useful? Yes. It represents a mammoth task in collating information on 600+ parasites into a book that fits (just) into your jacket pocket. It is primarily a reference book, a very good one at that, and I will certainly be making much use of it in the future.

## Competing interests

The author declares that he has no competing interests.

